# Biocatalytic Synthesis of Novel Partial Esters of a Bioactive Dihydroxy 4-Methylcoumarin by *Rhizopus oryzae* Lipase (ROL)

**DOI:** 10.3390/molecules21111499

**Published:** 2016-11-09

**Authors:** Vinod Kumar, Divya Mathur, Smriti Srivastava, Shashwat Malhotra, Neha Rana, Suraj K. Singh, Brajendra K. Singh, Ashok K. Prasad, Anjani J. Varma, Christophe Len, Ramesh C. Kuhad, Rajendra K. Saxena, Virinder S. Parmar

**Affiliations:** 1 Centre of Innovative and Applied Bioprocessing (CIAB), SAS Nagar, Mohali 160 071, Punjab, India; vinod_biodiesel@yahoo.co.in; 2 Department of Microbiology, University of Delhi South Campus, Benito Juarez Road, New Delhi 110 021, India; Kuhad85@gmail.com (R.C.K.); rksmicro@yahoo.co.in (R.K.S.); 3 Bioorganic Laboratory, Department of Chemistry, University of Delhi, Delhi 110 007, India; dmchem05@gmail.com (D.M.); smritichem10@gmail.com (S.S.); shashwatmalhotra@gmail.com (S.M.); neharana256@gmail.com (N.R.); surajsingh565656@gmail.com (S.K.S.); chemquestbk@gmail.com (B.K.S.); ashokenzyme@gmail.com (A.K.P.); 4 Department of Chemistry, Daulat Ram College, University of Delhi, Delhi 110 007, India; 5 Department of Chemistry, Kirori Mal College, University of Delhi, Delhi 110 007, India; 6 School of Chemical Sciences, Central University of Haryana, Mahendragarh, Haryana 123 029, India; anjanikumar@cuh.ac.in; 7 Sorbonne Universités, Université de Technologie de Compiègne, EA4297 Transformations Chimiques de la Matière Renouvelable, F-60203 Compiègne CEDEX, France; christophe.len@utc.fr; 8 Institute of Advanced Sciences, 410 Faunce Corner Mall Road, Dartmouth, MA 02747, USA

**Keywords:** *Rhizopus oryzae*, lipase, 7,8-Dihydroxy-4-methylcoumarin, regioselectivity, acylation

## Abstract

Highly regioselective acylation has been observed in 7,8-dihydroxy-4-methylcoumarin (DHMC) by the lipase from *Rhizopus oryzae* suspended in tetrahydrofuran (THF) at 45 °C using six different acid anhydrides as acylating agents. The acylation occurred regioselectively at one of the two hydroxy groups of the coumarin moiety resulting in the formation of 8-acyloxy-7-hydroxy-4-methylcoumarins, which are important bioactive molecules for studying biotansformations in animals, and are otherwise very difficult to obtain by only chemical steps. Six monoacylated, monohydroxy 4-methylcoumarins have been biocatalytically synthesised and identified on the basis of their spectral data and X-ray crystal analysis.

## 1. Introduction

**Figure 1:**
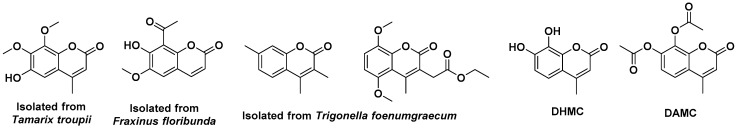
Biologically-important coumarins.

Coumarins (2*H*-1-benzopyran-2-ones) are versatile and a structurally-diverse class of natural products essentially found in the family *Rutaceae* and *Umbelifferae*. Owing to their plethora of pharmacological activities [1,2], coumarins have been a subject of extensive research over many decades [3,4]. In recent years, our laboratories have been actively engaged in studying the synthesis and biological activities of a wide range of coumarin derivatives [5,6,7,8]. We have previously isolated 8-acetyl-7-hydroxy-6-methoxycoumarin and 8-methoxycoumarin from *Fraxinus floribunda* leaves [9], trigoforin (3,4,7-trimethylcoumarin) and trigocoumarin [3-(ethoxycarbonyl)methyl-4-methyl-5,8-dimethoxycoumarin] from *Trigonella foenumgraecum* stems [10,11], and troupin (4-methyl-6-hydroxy-7,8-dimethoxycoumarin) from *Tamarix troupii* [12] (Figure 1).

Protein acetylation, largely acetyl coenzyme A (CoA)-dependent, is one of the prominent post-translational modifications of proteins that regulate several cellular processes [13]. Acetyl CoA-independent protein acetylation is also known, but is restricted to the action of aspirin-like drugs that would readily acetylate cyclooxygenase resulting in the inhibition of prostaglandin synthesis and, thus, induce anti-inflammatory effects [14]. Through extensive studies, we identified a microsomal enzyme, protein transacetylase (TAase) in mammalian cells and tissues, catalysing the transfer of acetyl groups from 7,8-diacetoxy-4-methylcoumarin (DAMC) to certain receptor proteins, viz*.*, cytosolic glutathione *S*-transferase (GST), cytochrome P-450-linked mixed function oxidases (MFO), Nicotinamide Adenine Dinucleotide Phosphate (NADPH) cytochrome c-reductase, protein kinase C (PKC), nitric oxide synthase (NOS), and recombinant glutamine synthetase (rGlnA1) of *Mycobacterium tuberculosis*, resulting in the modulation of their catalytic activities and associated physiological effects [15,16,17,18,19,20]. The rat liver and human placental microsomal TAase was later identified as calreticulin, a calcium-binding protein in the lumen of the endoplasmic reticulum and, consequently, the acetyltransferase function of calreticulin was appropriately termed calreticulin transacetylase (CRTAase) [21,22,23,24].

**Figure 2:**
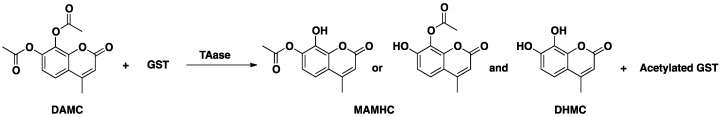
TAase catalyzed reaction.

Furthermore, we had earlier demonstrated that buffalo liver TAase-mediated inhibition of GST by DAMC was due to the acetylation of six lysine residues, viz., Lys-51, -82, -124, -181, -191, -210, and N-terminus proline residues, as shown by Matrix-Assisted Laser Desorption Ionization–Time of Flight (MALDI-TOF) and Liquid Chromatography–Mass Spectrometry (LC/MS/MS) studies [25]. It was also inferred that the C-7 acetoxy group of DAMC helps the C-8 acetoxy group to orient itself most favourably towards the oxygen heteroatom of the coumarin moiety and this phenomenon, presumably controls the TAase-mediated transfer of acetyl groups to the functional proteins [26]. An interesting observation was the action of TAase and DAMC on liver cytosolic GST, which resulted in the formation of a monoacetoxy, monohydroxy 4-methylcoumarin (MAMHC), and 7,8-dihydroxy-4-methylcoumarin (DHMC), although the former was the major metabolite (Figure 2) [27]. This substantiates the fact that acetyl groups are preferentially transferred by TAase from a particular acetoxy group of DAMC to the receptor protein leading to the accumulation of MAMHC. Nonetheless, the position of mono-deacetylation in DAMC could not be ascertained. Thus, to identify and compare the monoacetoxy, monohydroxy 4-methylcoumarin (MAMHC), it was important to synthesize it chemically. However, its chemical synthesis is still not known, since regioselective chemical mono-acylation of polyhydric phenols often requires a series of tedious steps leading to low yields of the required product.

Lipases provide an alternative, greener, and expedient route for the selective protection and deprotection of amino, hydroxyl, and carboxyl groups in a wide range of carbohydrates, nucleosides, steroids, and various other classes of organic molecules. Enzyme-mediated preparation of partial esters of multi-hydroxy compounds, such as polyphenols, has also been extensively explored owing to the stereo- and regioselective preferences of proteases [28,29,30,31,32]. We have reported efficient and straightforward regioselective protection/deprotection reactions on hydroxyl groups in an array of different classes of substrates using a miscellany of lipases, viz*.* porcine pancreatic lipase (PPL), *Candida antarctica* lipase-B (Novozyme^®^-435), lipase Amano PS, *Pseudomonas* sp. lipase, *Candida rugosa* lipase (CRL), and *Thermomyces lanuginosus* lipase (Lipozyme^®^ TL IM) in dry organic solvents [33,34,35,36].

As part of our ongoing research programme towards screening of various lipases/esterases for carrying out selective protection/deprotection of biologically-active scaffolds, and also to identify the MAMHC formed by the action of TAase, we have explored the possibility of using lipases for selective mono-acylation of 7,8-dihydroxy-4-methylcoumarin (Figure 2).

## 2. Results and Discussion

**Scheme 1:**
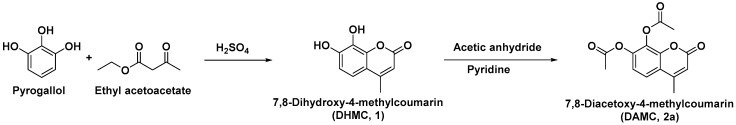
Synthesis of 7,8-dihydroxy-4-methylcoumarin and 7,8-diacetoxy-4-methylcoumarin.

In the present investigation, we have identified *Rhizopus oryzae* lipase (ROL)*,* a fungal lipase that assisted in the formation of monoacyloxy, monohydroxy 4-methylcoumarins from the biocatalytic acylation of 7,8-dihydroxy-4-methylcoumarin (DHMC) with different acid anhydrides (Scheme 1). It has been observed that greater selectivity and feasibility is achieved in the enzymatic deacylation reactions, particularly in the case of polyphenols [30,31]. With only a few reports dealing with the lipase-catalyzed regioselective acylation of phenolic hydroxy groups, such substrates seem to be difficult targets for acylation, notably because a phenolic hydroxyl group is less nucleophilic and is also known to adversely affect the enzymatic activity [37]. Therefore, we first conducted deacylation studies on 7,8-diacetoxy-4-methylcoumarin (DAMC, Figure 1). The enzymatic substrate was prepared by the well-known Pechmann condensation of pyrogallol with ethyl acetoacetate yielding DHMC, followed by its acetylation using acetic anhydride in pyridine (Scheme 1) [38].

**Scheme 2:**
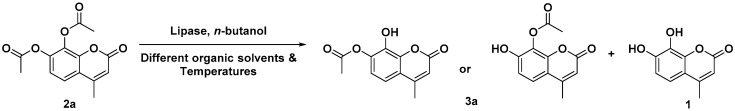
Enzymatic deacetylation studies on 7,8-diacetoxy-4-methylcoumarin (**2a**).

Interestingly, when we investigated the selective deacetylation of DAMC using lipases, viz., Lipozyme^®^ TL IM, PPL, CRL, and ROL in the presence of *n*-butanol as an acyl scavenger in different organic solvents, it did not yield any appreciable amount of any deacetylated product (Scheme 2). Monoacetoxy, monohydroxy 4-methylcoumarin (MAMHC) was formed in very low yields with DHMC as the major product in the case of Novozyme^®^-435 in THF at 50 °C.

Consequently, we tried acylation of 7,8-dihydroxy-4-methylcoumarin (DHMC, **1**) using various acid anhydrides and were successful in obtaining the monoacyloxy, monohydroxy 4-methylcoumarins exclusively. Five different lipases, viz., Novozyme^®^-435, Lipozyme^®^ TL IM, PPL, CRL, and ROL were screened for regioselective acylation of one of the two hydroxyl groups in 7,8-dihydroxy-4-methylcoumarin (**1**) using acetic anhydride in different organic solvents, i.e., THF, 1,4-dioxane, diisopropyl ether (DIPE), dimethylformamide (DMF), toluene, and acetonitrile at different temperatures (35–60 °C) and at 200 rpm in an incubator shaker. No appreciable conversion was observed during acylation reaction mediated by Lipozyme^®^ TL IM, PPL, or CRL in any of the six organic solvents. Novozyme^®^-435 showed limited regioselectivity towards DHMC in THF at 40 °C for acylation with the concomitant formation of MAMHC and DAMC. Additionally, the reaction was rather slow and did not lead to complete consumption of DHMC even after two days and at higher temperatures.

**Scheme 3:**
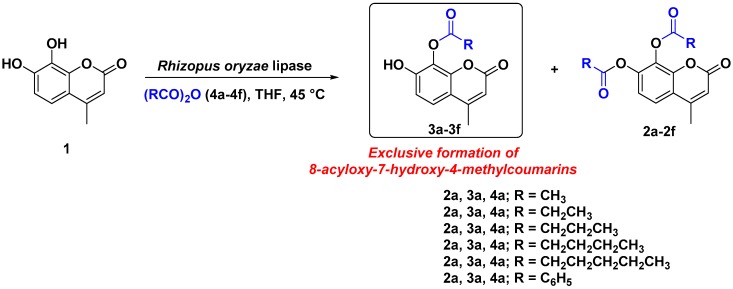
*Rhizopus oryzae* lipase-mediated regioselective acylation of DHMC (**1**) yielding exclusive formation of 8-acyloxy-7-hydroxy-4-methylcoumarins **3a**–**3f**.

It has been established that fungal lipases have benefits over bacterial lipases due to their low cost of extraction, thermal and pH stability, substrate specificity, and activity in organic solvents [39]. Keeping this in mind, the regioselective acylation of 7,8-dihydroxy-4-methylcoumarin (**1**) using acetic anhydride (**4a**) was attempted using lipase from *Rhizopus oryzae,* available in our laboratory culture collection, in different organic solvents, i.e., THF, 1,4-dioxane, DIPE, DMF, toluene, and acetonitrile, at different temperatures. *Rhizopus oryzae* lipase (ROL) in THF at 45 °C showed the best result with the formation of a new product with higher R*_f_* value than the starting substrate and the disappearance of the starting compound, as indicated by TLC examination in 18 h (Scheme 3). Though, some amount of DAMC (**2a**) was also isolated (but only) in traces, it is certain to say that *Rhizopus oryzae* lipase (ROL) displayed better regioselectivity, as well as better catalytic activity.

To explore the enzyme selectivity and reaction rate for different acyl groups, different acid anhydrides, *viz.*, propanoic (**4b**), butanoic (**4c**), pentanoic (**4d**), hexanoic (**4e**), and benzoic anhydride (**4f**) were evaluated towards the regioselective acylation reaction for selective protection of hydroxyl groups in 7,8-dihydroxy-4-methylcoumarin (**1**) using ROL in THF at 45 °C (Scheme 3, Table 1). On completion of the acylation reaction, as indicated by the disappearance of the starting compound on TLC, the enzyme was filtered off and the crude products thus obtained were purified using column chromatography. The mono-acylated coumarin derivatives **3a**–**3f** were obtained in 30%–82% isolated yields.

**Figure 3:**
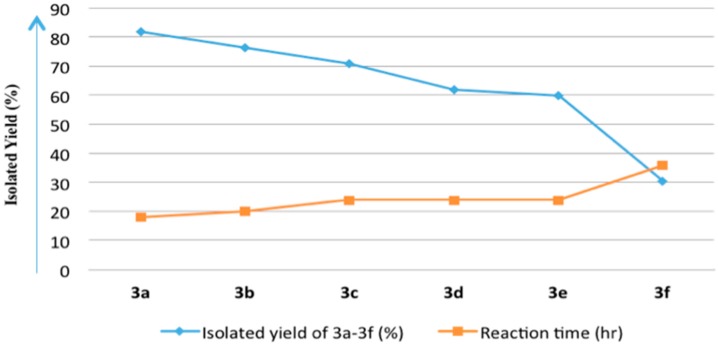
Conversion of 7,8-dihydroxy-4-methylcoumarin (**1**) to 8-acyloxy-7-hydroxy-4-methylcoumarins **3a**–**3f** using *Rhizopus oryzae* lipase (ROL).

It was observed that by increasing the acyl chain lengths, the reaction time increases. It was also noticed that the percentage yield of mono-acylated product **3a**–**3f** decreased with the increase in the acyl chain length (Figure 3). Further, it was contemplated that the increase in the acyl chain length and reaction time also led to the increase in the formation of diacylated coumarin derivative **2a**–**2f**. We found acetic anhydride (**4a**) as the best acylating agent, among others, in terms of reaction time, yield, and percentage conversion to the desired regioselectively-acylated products (Table 1, Figure 3).

Some amounts of the diacyloxycoumarins **2b**–**2e** were also observed, but were not isolated due to low yields. In the case of benzoic anhydride (**4f**), the reaction was too slow to be of any particular application with the formation of the mono-benzoyl coumarin derivative **3f** in 30.5% yield after 36 h, together with the formation of the dibenzoyl coumarin derivative **2f** in 59% isolated yield (Table 1). Thus, it can be inferred that aromatic acid anhydride did not show good selectivity, primarily due to steric hindrance. Along the same lines, alkyl groups having longer chain lengths also lead to poor selectivity.

**Figure 4:**
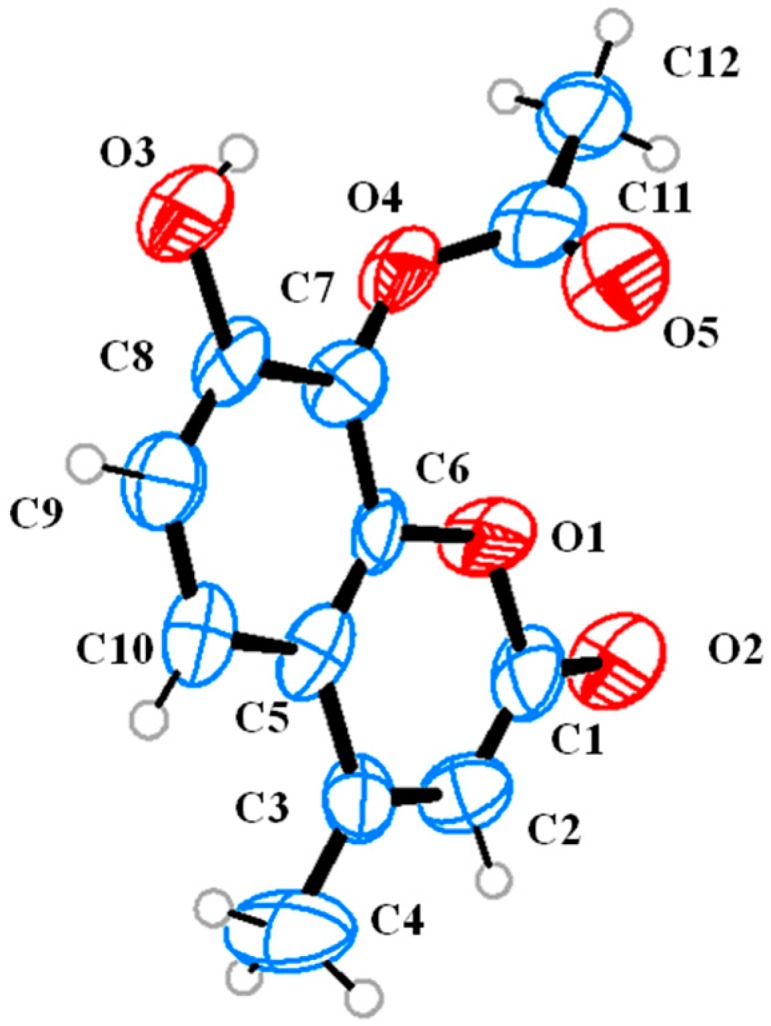
ORTEP diagram showing crystallographic atom numbering and solid-state conformation for 8-acetoxy-7-hydroxy-4-methylcoumarin (**3a**; CCDC-1508122). Crystallographic data have been deposited with Cambridge Crystallographic Data.

The site of acylation was confirmed by single X-ray crystal analysis of compound **3a** synthesized by biocatalytic acetylation of 7,8-dihydroxy-4-methylcoumarin with acetic anhydride and ROL (Table S1, Supplementary Materials). The structure of **3a** was found to be 8-acetoxy-7-hydroxy-4-methylcoumarin which established the fact that the enzymatic acylation occurred regioselectively at the C-8 hydroxyl group instead of the C-7 hydroxyl group in the presence of the ROL (Figure 4). It was previously reported by us that, in buffalo liver, TAase mediated inhibition of GST by DAMC, the C-7 acetoxy group helps the C-8 acetoxy group to orient itself towards the oxygen heteroatom of the coumarin leading to the formation of MAMHC [16,26]. Selective formation of 8-acyloxy-7-hydroxy-4-methylcoumarin by ROL mediated acylation corroborates this observation.

This unique selectivity exhibited by the *Rhizopus oryzae* lipase (ROL) in discriminating the esterification of one out of two phenolic hydroxyl groups of similar reactivity is not feasible to achieve by chemical methodologies. All attempts in our laboratories during the last three decades by any biocatalytic or chemical sequence of reactions have been futile in obtaining the partial esters of di- or trihydric phenolic compounds which we needed for synthesizing the bioactive analogues of naturally-occurring flavonoids, coumarins, xanthones, or isoflavonoids having interesting biological activities in cancer, antioxidant, and antiviral research. Thus, the results presented in this study can find wide applications in various fields of organic chemistry and medicinal chemistry.

## 3. Experimental Section

### 3.1. Reagents and Solvents

All reagents used were of commercial grade. Solvents were dried and purified using standard techniques. *Rhizopus oryzae,* obtained from our laboratory culture collection was maintained on potato dextrose agar (PDA). The purified lipase gives a single band on SDS PAGE with a mol. wt. of 30 ± 1 kDa. ^1^H-NMR (400 MHz) and ^13^C-NMR (100.5 MHz) were recorded in DMSO. Chemical shifts are reported in parts per million (ppm). The chemical shift values are on the δ scale and the coupling constants (*J*) are in Hz. Mass spectra were recorded on a KC ESI 455-TOF mass spectrometer (Micromass, Manchester, UK). The IR spectra were recorded on a Perkin-Elmer model 2000 FT-IR spectrometer (Perkin Elmer Co., Massachusetts, MA, USA) by making a KBr disc for solid samples and thin film for oils. X-ray diffraction data was collected on an Oxford XCalibur CCD diffractometer (Oxford Diffraction, Abingdon, Oxfordshire, UK) using graphite-monochromated Cu Kα radiation (λ = 0.7107 Å) at a temperature 298 K. The structure was solved by direct methods using SIR-92 (Semi-Invariants Representation) [40] and refined by the full-matrix method (SHELXL-2016/4) [41]. The reactions were monitored by thin layer chromatography (TLC) on aluminium plates coated with silica-gel 60F_254_ (Merck KGaA, Darmstadt, Germany). UV radiation and iodine were used as the visualizing agents. Column chromatography was performed on silica gel (100–200 mesh, Merck KGaA).

### 3.2. Lipase Production Conditions

Production of lipase was carried out in 2 L Erlenmeyer flasks containing 400 mL of optimized production medium (% *w/v*: soybean oil (emulsified with 0.2% gum acacia), 1.5; cane molasses, 1.0; maize starch, 1.0; soybean meal, 2.0; CaCl_2_∙2H_2_O, 0.10; KH_2_PO_4_, 0.50, pH 9.0) inoculated with 8 × 10^7^ spores and incubated at 30 °C, 200 rpm for 96 h. The culture broth was subjected to filtration and centrifugation to remove the biomass, followed by a four-fold concentration by ultrafiltration using 10 kDa cellulose acetate membranes (Millipore Merck, Darmstadt, Germany). This partially purified and concentrated lipase was precipitated using the protocol of Shu et al. [42] and lyophilized. This preparation had a lipase activity of 3.6 U/mg and was used as such (in the native form) in the present study.

### 3.3. Determination of Lipase Activity

The lipase activity of the native lipase was measured by the procedure described by Winkler and Stuckmann [43]. One unit (U) of enzyme activity was defined as the amount of enzyme, which liberates 1 µmol of *p*-nitrophenol from *p*-nitrophenyl acetate per minute under assay conditions.

### 3.4. Synthesis

#### Regioselective *Rhizopus oryzae* Lipase (ROL)-Mediated Acylation Reaction on 7,8-Dihydroxy-4-methylcoumarin (**1**).

To a dried round bottom flask, 7,8-dihydroxy-4-methylcoumarin (**1**) (2 mmol) was added, followed by the addition of dry THF (25 mL). To the solution, acid anhydride **4a**–**4f** (2 mmol) was slowly added with continuous stirring. Further, lipase from *Rhizopus oryzae* (500 mg) was carefully added into the stirring reaction mixture. The reaction mixture was allowed to shake in an incubator shaker at 45 °C and was regularly monitored on TLC using 1:9 methanol:chloroform as solvent system. On completion of the reaction, the enzyme was filtered off and washed twice with methanol (2 × 10 mL). The solvent was evaporated in vacuo and the crude 8-acyloxy-7-hydroxy-4-methylcoumarins **3a**–**3f** thus obtained were purified using column chromatography with chloroform/methanol as the eluent.

*8-Acetoxy-7-hydroxy-4-methylcoumarin* (**3a**): White solid; 82.0% yield; m.p. 234–235 °C. IR (film) v_max_ (cm^−1^): 3103, 1774, 1692, 1605, 1442, 1370, 1279, 1222, 1171. ^1^H-NMR (400 MHz, DMSO-*d*_6_) δ: 10.78 (s, 1H, OH), 7.50 (d, *J* = 8.79 Hz, 1H, ArH), 6.93 (d, *J* = 8.79 Hz, 1H, ArH), 6.17 (s, 1H, H-3), 2.37 (s, 3H, C-4 CH_3_), 2.34 (s, 3H, COCH_3_). ^13^C-NMR (100.5 MHz, DMSO-*d*_6_) δ: 168.25, 159.43, 154.01, 152.90, 146.85, 125.05, 123.05, 112.97, 112.64, 110.53, 20.31, 18.27. HRMS (ESI): *m*/*z* calcd. for C_12_H_10_O_5_ + Na^+^ [M + Na]^+^: 257.0420. Found 257.0421.

*7-Hydroxy-8-propanoyloxy-4-methylcoumarin* (**3b**): White solid; 76.5% yield; m.p. 190–191 °C. IR (film): ν_max_ (cm^−1^); 3140, 1765, 1693, 1601, 1453, 1393, 1360, 1281, 1196, 1177. ^1^H-NMR (400 MHz, DMSO-*d*_6_) δ: 10.73 (s, 1H, OH), 7.47 (d, *J* = 8.68 Hz, 1H, ArH), 6.91 (d, *J* = 8.68 Hz, 1H, ArH), 6.16 (s, 1H, H-3), 2.64 (q, *J* = 7.36 Hz, 2H, COCH_2_CH_3_), 2.39 (s, 3H, C-4 CH_3_), 1.14 (t, *J* = 7.32 Hz, 3H, COCH_2_CH_3_). ^13^C-NMR (100.5 MHz, DMSO-*d*_6_) δ: 171.58, 159.41, 153.89, 152.92, 146.77, 125.02, 122.88, 112.92, 112.43, 110.41, 26.47, 18.21, 9.00. HRMS (ESI): *m*/*z* calcd. for C_13_H_12_O_5_ + Na^+^ [M + Na]^+^: 271.0577. Found 271.0574.

*8-Butanoyloxy-7-hydroxy-4-methylcoumarin* (**3c**): Cream-coloured solid; 71.0% yield; m.p. 145–146 °C. IR (film): ν_max_ (cm^−1^), 3306, 2971, 2937, 2879, 1761, 1715, 1609, 1580, 1327, 1271, 1189, 1243, 1171, 1139. ^1^H-NMR (400 MHz, DMSO-*d*_6_) δ: 10.77 (s, 1H, OH), 7.50 (d, *J* = 8.80 Hz, 1H, ArH), 6.93 (d, *J* = 8.80 Hz, 1H, ArH), 6.18 (s, 1H, H-3), 2.62 (t, *J* = 7.32 Hz, 2H, COCH_2_CH_2_CH_3_), 2.39 (s, 3H, C-4 CH_3_), 1.66–1.76 (m, 2H, COCH_2_CH_2_CH_3_), 1.01 (t, *J* = 7.32 Hz, 3H, COCH_2_CH_2_CH_3_). ^13^C-NMR (100.5 MHz, DMSO-*d*_6_) δ: 170.63, 159.27, 153.84, 152.91, 146.76, 125.06, 122.86, 112.86, 112.50, 110.41, 39.08, 18.19, 18.10, 13.32. HRMS (ESI): *m*/*z* calcd. for C_14_H_14_O_5_ + Na^+^ [M + Na]^+^: 285.0733. Found 285.0730.

*7-Hydroxy-8-pentanoyloxy-4-methylcoumarin* (**3d**): Light gray solid; 62.0% yield; m.p. 143–145 °C. IR (film): ν_max_ (cm^−1^), 3139, 2937.29, 1764, 1709, 1602, 1515, 1447, 1366, 1273, 1212, 1134, 1093. ^1^H-NMR (400 MHz, DMSO-*d*_6_) δ: 10.77 (s, 1H, OH), 7.48 (d, *J* = 8.70 Hz, 1H, ArH), 6.91 (d, *J* = 8.70 Hz, 1H, ArH), 6.16 (s, 1H, H-3), 2.63 (t, *J* = 7.30 Hz, 2H, COCH_2_CH_2_CH_2_CH_3_), 2.36 (s, 3H, C-4 CH_3_), 2.15–2.19 (m, 2H, COCH_2_CH_2_CH_2_CH_3_), 1.43–1.45 (m, 2H, COCH_2_CH_2_CH_2_CH_3_), 0.91 (t, *J* = 7.33 Hz, 3H, COCH_2_CH_2_CH_2_CH_3_). ^13^C-NMR (100.5 MHz, DMSO-*d*_6_) δ: 174.56, 170.77, 159.28, 153.84, 152.93, 125.03, 122.87, 112.88, 110.43, 33.38, 32.79, 26.64, 21.48, 18.20, 13.64. HRMS (ESI): *m*/*z* calcd. for C_15_H_16_O_5_ + Na^+^ [M + Na]^+^: 299.0890. Found 299.0889.

*8-Hexanoyloxy-7-hydroxy-4-methylcoumarin* (**3e**): Light yellow solid; 60.0% yield; m.p. 139–141 °C. IR (film) ν_max_ (cm^−1^): 3166, 2932, 1777, 1698, 1602, 1515, 1440, 1369, 1172, 1128. ^1^H-NMR (400 MHz, DMSO-*d*_6_) δ: 10.62 (s, 1H, OH), 7.36 (d, *J* = 9.16 Hz, 1H, ArH), 6.80 (d, *J* = 9.16 Hz, 1H, ArH), 6.04 (s, 1H, H-3), 2.50 (t, *J* = 7.32 Hz, 2H, COCH_2_CH_2_CH_2_CH_2_CH_3_), 2.25 (s, 3H, C-4 CH_3_), 1.52–1.59 (m, 2H, COCH_2_CH_2_CH_2_CH_2_CH_3_), 1.20–1.28 (m, 4H, COCH_2_CH_2_CH_2_CH_2_CH_3_), 0.75 (t, *J* = 6.88 Hz, 3H, COCH_2_CH_2_CH_2_CH_2_CH_3_). ^13^C-NMR (100.5 MHz, DMSO-*d*_6_) δ: 170.75, 159.25, 153.81, 152.90, 146.78, 125.01, 122.84, 112.85, 112.50, 110.43, 33.02, 30.47, 24.24, 21.83, 18.19, 13.80. HRMS (ESI): *m*/*z* calcd. for C_16_H_18_O_5_ + H^+^ [M + H]^+^: 291.1227. Found 291.1214.

*8-Benzoyloxy-7-hydroxy-4-methylcoumarin* (**3f**): Cream-coloured solid; 30.5% yield; m.p. 201–203 °C. IR (film): ν_max_ (cm^−1^) 3308, 2927, 1719, 1735, 1608, 1586, 1380, 1258, 1234. ^1^H-NMR (400 MHz, DMSO-*d*_6_) δ: 10.96 (s, 1H, OH), 8.16 (d, *J* = 7.32 Hz, 1H, ArH), 7.92 (d, *J* = 7.32 Hz, 1H, ArH), 7.76 (m, 1H, ArH), 7.46–7.65 (m, 3H, ArH), 6.99 (d, *J* = 8.80 Hz, 1H, ArH), 6.18 (s, 1H, H-3), 2.40 (s, 3H, C-4 CH_3_). ^13^C-NMR (100.5 MHz, DMSO-*d*_6_) δ: 167.34, 163.58, 159.30, 153.95, 153.08, 146.87, 134.35, 132.87, 130.05, 129.27, 129.10, 128.57, 128.20, 123.16, 113.02, 112.60, 110.46, 18.24. HRMS (ESI): *m*/*z* calcd. for C_17_H_12_O_5_ + Na^+^ [M + Na]^+^: 319.0577. Found 319.0571.

## 4. Conclusions

In conclusion, we have chemo-enzymatically synthesized a series of novel, hitherto unknown, 8-acyloxy-7-hydroxy-4-methylcoumarins in a regioselective fashion using the *Rhizopus oryzae* lipase (ROL) in THF at 45 °C from 7,8-dihydroxy-4-methylcoumarin (DHMC), which, if not impossible, are very tedious and cumbersome to synthesize via chemical routes. It was observed that with an increase in the size of the alkyl group of the acylating agent, the reaction time increased with a decrease in the isolated yield of the desired product. The acylation selectively occurred at the C-8 hydroxyl group, which was established using single X-ray crystal analysis.

## Figures and Tables

**Table 1:** *Rhizopus oryzae* lipase (ROL)-catalyzed regioselective formation of 8-acyloxy-7-hydroxy-4-methylcoumarins **3a**–**3f** in THF at 45 °C.

Entry	Acid Anhydride (4a–4f)	Isolated Yield of 8-Acyloxy-7-hydroxy-4-methylcoumarin (3a–3f) ^a^ (%)	7,8-Diacyloxy-4-methylcoumarin (2a–2f)	Reaction Time (h)
1	Acetic anhydride (**4a**)	**3a**/82.0	**2a**/Traces ^b^	18
2	Propanoic anhydride (**4b**)	**3b**/76.5	**2b**/<10% ^b^	20
3	Butanoic anhydride (**4c**)	**3c**/71.0	**2c**/<10% ^b^	24
4	Pentanoic anhydride (**4d**)	**3d**/62.0	**2d**/<20% ^b^	24
5	Hexanoic anhydride (**4e**)	**3e**/60.0	**2e**/<20% ^b^	24
6	Benzoic anhydride (**4f**)	**3f**/30.5	**2f**/59.0%	36

^a^ All of these reactions did not yield any product when performed in the absence of the lipase; ^b^ Products not isolated, results according to the TLC analysis.

## Supplementary Materials

Supplementary materials can be accessed at: http://www.mdpi.com/1420-3049/21/11/1499/s1. Crystallographic data for 8-acetoxy-7-hydroxy-4-methylcoumarin (**3a**; CCDC-1508122) have been deposited with the Cambridge Crystallographic Data, Table S1: Single crystal X-ray diffraction data of compound **3a**.

## References

[1] Macáková K., Řeháková Z., Mladěnka P., Karlíčková J., Filipský T., Říha M., Prasad A.K., Parmar V.S., Jahodář L., Pávek P. (2012). In vitro platelet antiaggregatory properties of 4-methylcoumarins. Biochimie.

[2] Kostova I., Bhatia S., Grigorov P., Balkansky S., Parmar V.S., Prasad A.K., Saso L. (2011). Coumarins as antioxidants. Curr. Med. Chem..

[3] Medina F.G., Marrero J.G., Macías-Alonso M., González M.C., Córdova-Guerrero I., García A.G.T., Osegueda-Robles S. (2015). Coumarin heterocyclic derivatives: Chemical synthesis and biological activity. Nat. Prod. Rep..

[4] Gaudino E.C., Tagliapietra S., Martina K., Palmisano G., Cravotto G. (2016). Recent advances and perspectives in the synthesis of bioactive coumarins. RSC Adv..

[5] Arya A., Kumar V., Mathur D., Singh S., Brahma R., Singh R., Singh S., Sharma G.L., Parmar V.S., Prasad A.K. (2015). Synthesis of potential bioactive novel 7-[2-hydroxy-3-(1,2,3-triazol-1-yl)propyloxy]-3-alkyl-4-methylcoumarins. J. Heterocycl. Chem..

[6] Mathur D., Prasad A.K., Cameron T.S., Jha A. (2014). Novel approach to 3,3-dimethyl-4-morpholino-3,4-dihydrocoumarins via hetero-Diels-Alder reaction. Tetrahedron.

[7] Joshi R., Kumar A., Manral S., Sinha R., Arora S., Sharma A., Goel S., Kalra N., Chatterji S., Dwarakanath B.S. (2013). Calreticulin transacetylase mediated upregulation of thioredoxin by 7,8-diacetoxy-4-methylcoumarin enhances the antioxidant potential and the expression of vascular endothelial growth factor in peripheral blood mononuclear cells. Chem. Biol. Interact..

[8] Kumar A., Singh B.K., Sharma N.K., Gyanda K., Jain S.K., Tyagi Y.K., Baghel A.S., Pandey M., Sharma S.K., Prasad A.K. (2006). Specificities of acetoxy derivatives of coumarins, biscoumarins, chromones, flavones, isoflavones and xanthones for acetoxy drug: Protein transacetylase. Eur. J. Med. Chem..

[9] Nagarajan G., Rani U., Parmar V.S. (1980). Coumarins from *Fraxinus floribunda* leaves. Phytochemistry.

[10] Khurana S.K., Krishnamoorthy V., Parmar V.S., Sanduja R., Chawla H.L. (1982). 3,4,7-Trimethylcoumarin from *Trigonella foenum-graecum* stems. Phytochemistry.

[11] Parmar V.S., Jha H.N., Sanduja S.K., Sanduja R. (1981). Trigocoumarin—A new coumarin from *Trigonella foenumgraecum*. Z. Naturforsch.

[12] Parmar V.S., Rathore J.S., Singh S., Jain A.K., Gupta S.R. (1985). Troupin, a 4-methylcoumarin from *Tamarix troupii*. Phytochemistry.

[13] Allis C.D., Chicoine L.G., Richman R., Schulman G. (1985). Deposition related histone acetylation in micronuclei of conjugation tetrahymena. Proc. Natl. Acad. Sci. USA.

[14] Vane J.R. (1971). Inhibition of prostaglandin synthesis as a mechanism of action for aspirin-like drugs. Nat. (New Biol.).

[15] Raj H.G., Parmar V.S., Jain S.C., Kohli E., Tyagi Y.K., Wengel J., Olsen C.E. (2000). Assay and characterization of 7,8-diacetoxy-4-methylcoumarin-protein transacetylase from rat liver microsomes based on the irreversible inhibition of cytosolic Glutathione *S*-transferase. Bioorg. Med. Chem..

[16] Singh I., Kohli E., Raj H.G., Gyanda K., Jain S.K., Tyagi Y.K., Gupta G., Kumari R., Kumar A., Pal G. (2002). Mechanism of biochemical action of substituted 4-methylbenzopyran-2-ones. Part 9: Comparison of acetoxy 4-methylcoumarins and other polyphenolic acetates reveal the specificity to acetoxy drug: Protein transacetylase for pyran carbonyl group in proximity to the oxygen heteroatom. Bioorg. Med. Chem..

[17] Khurana P., Kumari R., Vohra P., Kumar A., Seema, Gupta G., Raj H.G., Dwarakanath B.S., Parmar V.S., Saluja D. (2006). Acetoxy drug: Protein transacetylase catalyzed activation of human platelet nitric oxide synthase by polyphenolic peracetates. Bioorg. Med. Chem..

[18] Raj H.G., Parmar V.S., Jain S.C., Goel S., Singh A., Tyagi Y.K., Jha H.N., Olsen C.E., Wengel J. (1999). Mechanism of biochemical action of substituted 4-methylbenzopyran-2-ones. Part 4. Hyperbolic activation of rat liver microsomal NADPH cytochrome C reductase by the novel acetylator 7,8-diacetoxy-4-methylcoumarin. Bioorg. Med. Chem..

[19] Raj H.G., Parmar V.S., Jain S.C., Goel S., Singh A., Gupta K., Rohil V., Tyagi Y.K., Jha H.N., Olsen C.E. (1998). Mechanism of biochemical action of substituted 4-methylbenzopyran-2-one. Part II: Mechanism-based inhibition of rat liver microsome-mediated aflatoxin B_1_-DNA binding by the candidate antimutagen 7,8-diacetoxy-4-methylcoumarin. Bioorg. Med. Chem..

[20] Baghela A.S., Tandona R., Gupta G., Kumar A., Sharma R.K., Aggarwal N., Kathuria A., Saini N.K., Bose M., Prasad A.K. (2011). Characterization of protein acyltransferase function of recombinant purified GlnA1 from *Mycobacterium tuberculosis*: A moon lighting property. Microbiol. Res..

[21] Raj H.G., Kumari R., Seema, Muralidhar K.M., Dwarkanath B.S., Rastogi R.C., Prasad A.K., Watterson A.C., Parmar V.S. (2006). Novel function of calreticulin: Characterization of calreticulin as transacetylase-mediating protein acetylation independent of acetyl CoA using PA. Pure Appl. Chem..

[22] Seema, Kumari R., Gupta G., Saluja D., Kumar A., Goel S., Tyagi Y.K., Gulati R., Vinocha A., Muralidhar K.M. (2007). Characterization of protein transacetylase from human placenta as a signalling molecule calreticulin using polyphenolic peracetates as acetyl donors. Cell Biochem. Biophys..

[23] Bansal S., Gaspari M., Raj H.G., Kumar A., Cuda G., Verheij E., Tyagi Y.K., Ponnan P., Rastogi R.C., Parmar V.S. (2008). Calreticulin transacetylase mediates the acetylation of nitric oxide synthase by polyphenolic acetates. Appl. Biochem. Biotechnol..

[24] Singh U., Kumar A., Sinha R., Manral S., Arora S., Ram S., Mishra R.K., Gupta P., Bansal S.K., Prasad A.K. (2010). Calreticulin transacetylase catalyzed modification of the TNF-α mediated pathway in the human peripheral blood mononuclear cells by PA. Chem. Biol. Interact..

[25] Kohli E., Gaspari M., Raj H.G., Parmar V.S., van der Greef J., Gupta G., Kumari R., Prasad A.K., Goel S., Pal G. (2002). Establishment of the enzymatic protein acetylation independent of acetyl CoA: Recombinant glutathione *S*-transferase 3–3 is acetylated by a novel membrane-bound transacetylase using 7,8-diacetoxy-4-methylcoumarin as acetyl donor. FEBS Lett..

[26] Raj H.G., Kohli E., Goswami R., Goel S., Rastogi R.C., Jain S.C., Wengal J., Olsen C.E., Parmar V.S. (2001). Mechanism of biochemical action of substituted 4-methylbenzopyran-2-ones. Part-8: Acetoxycoumarin: Protein transacetylase specificity for aromatic nuclear acetoxy groups in proximity to the oxygen heteroatom. Bioorg. Med. Chem..

[27] Kohli E., Gaspari M., Raj H.G., Parmar V.S., Sharma S.K., van der Greef J., Kumari R., Gupta G., Seema, Khurana P. (2004). Acetoxy drug: Protein transacetylase of buffaloe liver: Characterization and mass spectrometry of the acetylated protein product. Biochim. Biophys. Acta.

[28] Torres P., Poveda A., Jimenez-Barbero J., Ballesteros A., Plou F.J. (2010). Regioselective lipase-catalyzed synthesis of 3-*O*-acyl derivatives of resveratrol and study of their antioxidant properties. J. Agric. Food Chem..

[29] Nicolosi G., Piattelli M., Sanfilippo C. (1992). Acetylation of phenols in organic solvent catalyzed by lipase from *Chromobacterium viscosum*. Tetrahedron.

[30] Miyazawa T., Hamada M., Morimoto R. (2016). *Candida antarctica* lipase B-catalyzed regioselective deacylation of dihydroxybenzenes acylated at both phenolic hydroxy groups. Can. J. Chem..

[31] Ciuffreda P., Casati S., Santaniello E. (2000). Regioselective hydrolysis of diacetoxynaphthalenes catalyzed by *Pseudomonas* sp. lipase in an organic solvent. Tetrahedron.

[32] Lambusta D., Nicolosi G., Patti A., Piatelli M. (1993). Enzyme-mediated regioprotection-deprotection of hydroxyl groups in (+)-catechin. Synthesis.

[33] Kumar R., Azim A., Kumar V., Sharma S.K., Prasad A.K., Howarth O.W., Olsen C.E., Jain S.C., Parmar V.S. (2001). Lipase-catalyzed chemo- and enantioselective acetylation of 2-alkyl/aryl-3-hydroxypropiophenones. Bioorg. Med. Chem..

[34] Kumar G., Dhawan A., Singh B.K., Sharma N.K., Sharma S.K., Prasad A.K., Van der Eycken E.V., Len C., Watterson A.C., Parmar V.S. (2015). Highly selective biocatalytic transesterification reactions on aryl 3-hydroxy-2-(hydroxymethyl)-2-methylpropanoates. Catal. Lett..

[35] Mathur D., Bohra K., Bhatia S., Kumar M., Verma P., Saxena R.K., Parmar V.S., Prasad A.K. (2011). Diastereoselective acetylation studies on 4-C-hydroxymethyl-1,2-*O*-isopropylidene-3-*O*-alkyl-β-l-*threo*-pentofuranose: Key precursor for biocompatible sugar-PEG copolymers. Trends Carbohydr. Res..

[36] Malhotra S., Calderón M., Prasad A.K., Parmar V.S., Haag R. (2010). Novel chemoenzymatic methodology for the regioselective glycine loading on polyhydroxy compounds. Org. Biomol. Chem..

[37] Rohn S., Rawel H.M., Kroll J. (2002). Inhibitory effects of plant phenols on the activity of selected enzymes. J. Agric. Food Chem..

[38] Pechmann H.V., Duisberg C. (1883). Ueber die Verbindungen der Phenole mit Acetessigäther. Ber. Dtsch. Chem. Ges..

[39] Singh A.K., Mukhopadhyay M. (2012). Overview of fungal lipase: A review. Appl. Biochem. Biotechnol..

[40] Altomare A., Cascarano G., Giacovazzo C., Guagliardi A., Burla M.C., Polidori G., Camalli M. (1999). *SIR*92—A program for automatic solution of crystal structures by direct methods. J. Appl. Cryst..

[41] Sheldrick G.M. (2008). A short history of SHELX. Acta Cryst..

[42] Shu C.H., Xu C.J., Lin G.C. (2006). Purification and partial characterization of a lipase from *Antrodia cinnamomea*. Process Biochem..

[43] Winkler U.K., Stuckmann M. (1979). Glucogen, hyaluronate and some other polysaccharides greatly enhance the formation of exolipase by *Serratia marcescens*. J. Bacteriol..

