# Supplementary material for: Biocatalytic Synthesis of Novel Partial Esters of a Bioactive Dihydroxy 4-Methylcoumarin by Rhizopus oryzae Lipase (ROL)
Source: Molecules. 2016 Nov 9;21(11):1499. doi: 10.3390/molecules21111499 (PMC6273029; doi:10.3390/molecules21111499)
Supplement: Supplementary file 1 [file molecules-21-01499-s001.pdf]

Supplementary Materials: Biocatalytic Synthesis of Novel Partial Esters of a Bioactive Dihydroxy 4-Methylcoumarin by *Rhizopus oryzae* Lipase (ROL)

Vinod Kumar, Divya Mathur, Smriti Srivastava, Shashwat Malhotra, Neha Rana, Suraj K. Singh, Brajendra K. Singh, Ashok K. Prasad, Anjani J. Varma, Christophe Len, Ramesh C. Kuhad, Rajendra K. Saxena and Virinder S. Parmar

1. Spectroscopic characterization: ^{1}H- & ^{13}C-NMR spectra of compounds 3a–3f

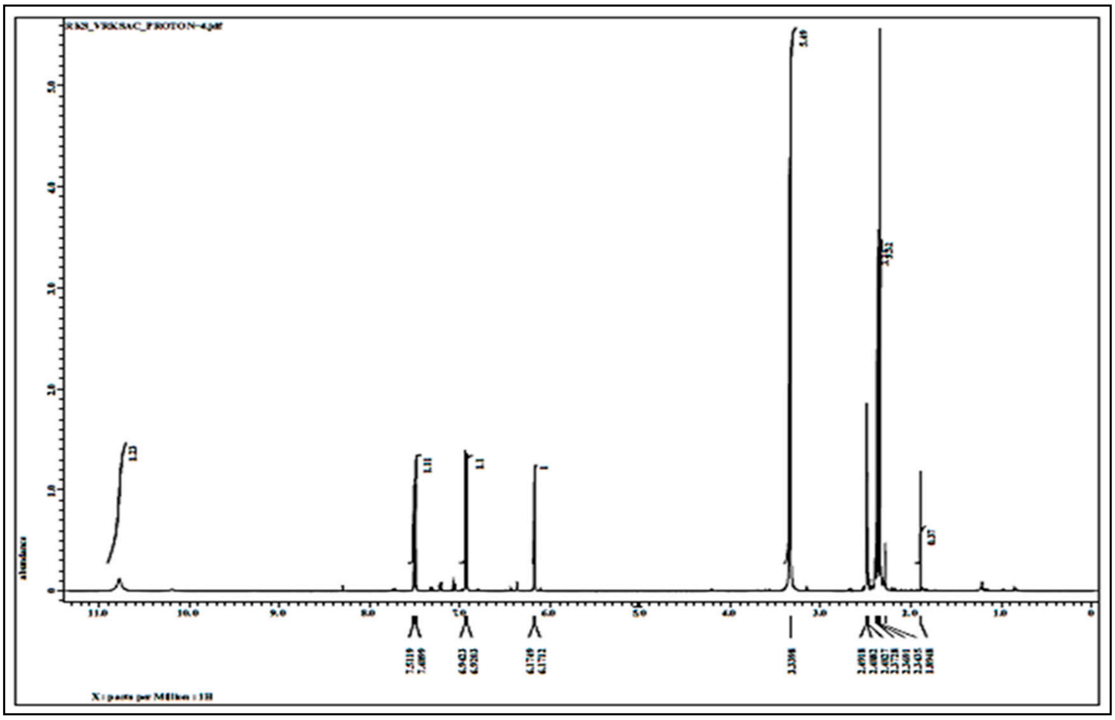

Figure S1. ^{1}H-NMR of 8-acetoxy-7-hydroxy-4-methylcoumarin (**3a**).

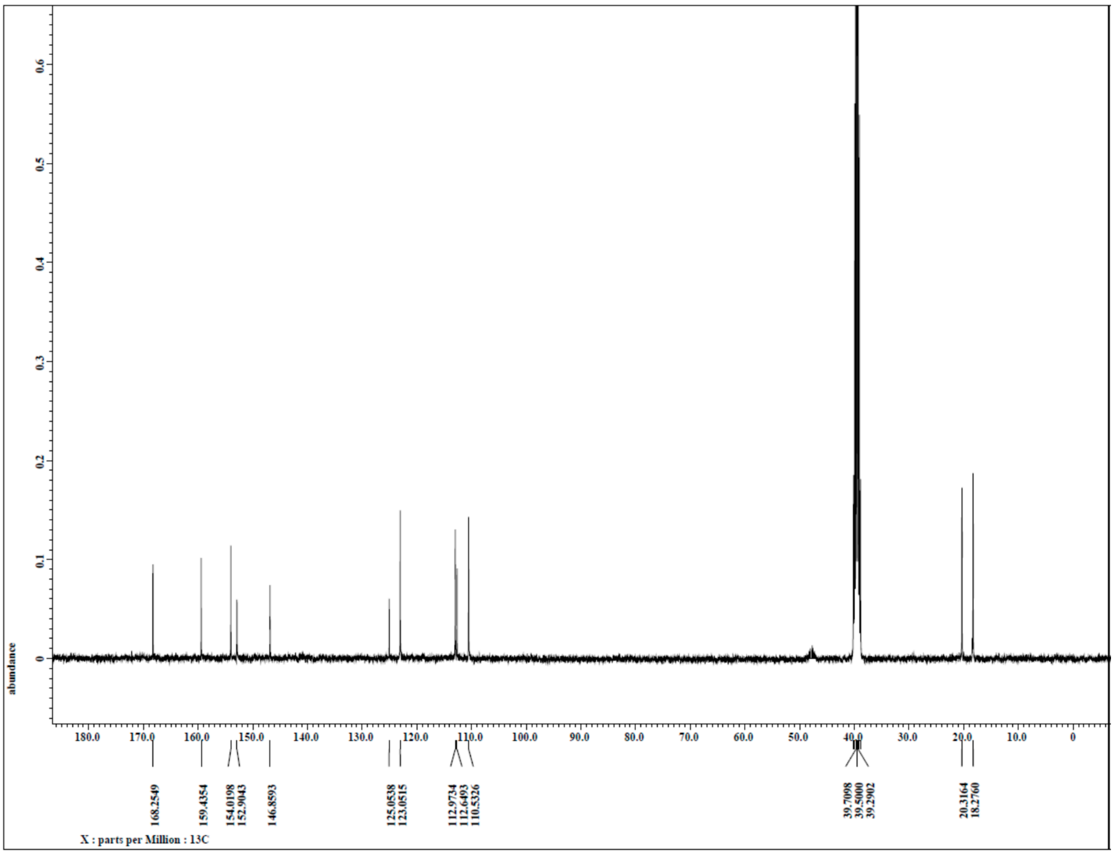

Figure S2. ^{13}C-NMR of 8-acetoxy-7-hydroxy-4-methylcoumarin (**3a**).

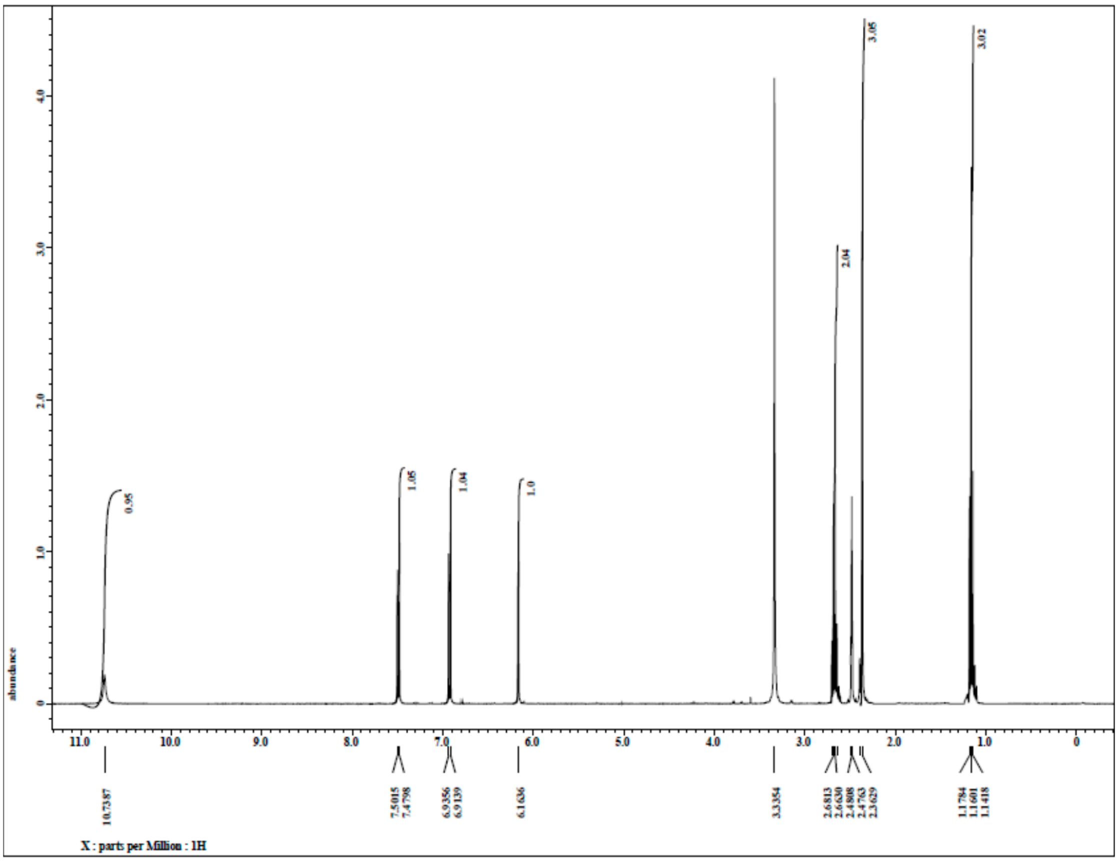

Figure S3. ^{1}H-NMR of 7-Hydroxy-8-propanoyloxy-4-methylcoumarin (**3b**).

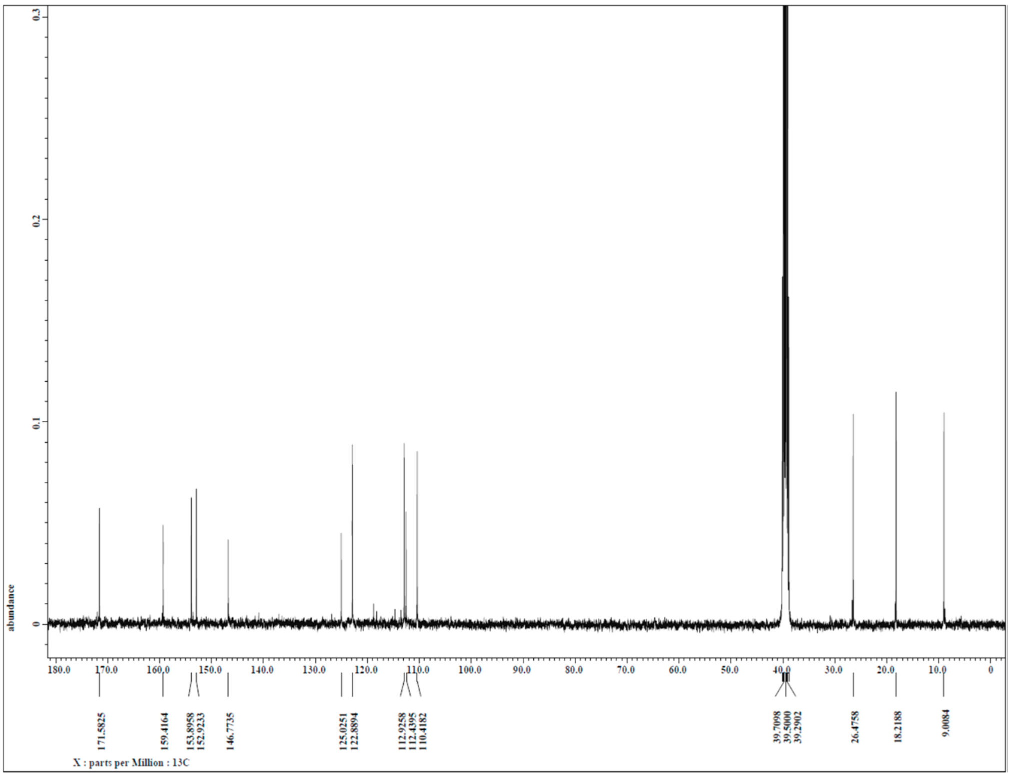

Figure S4. ^{13}C-NMR of 7-Hydroxy-8-propanoyloxy-4-methylcoumarin (**3b**).

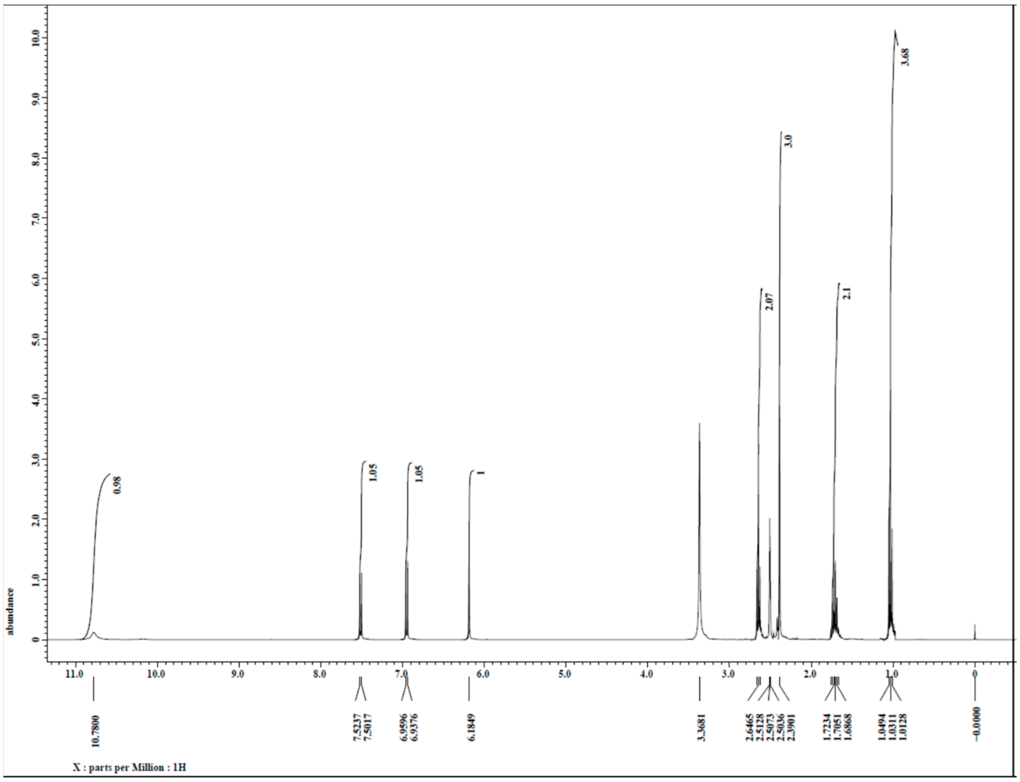

Figure S5. ^{1}H-NMR of 8-Butanoyloxy-7-hydroxy-4-methylcoumarin (**3c**).

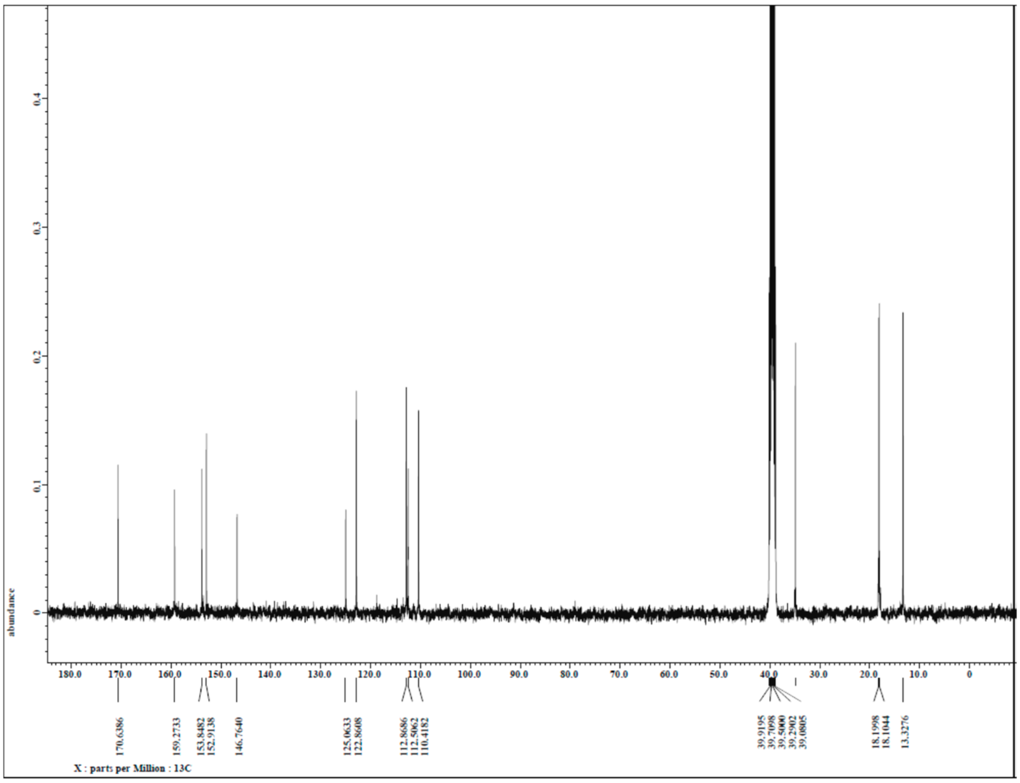

Figure S6. ^{13}C-NMR of 8-Butanoyloxy-7-hydroxy-4-methylcoumarin (**3c**).

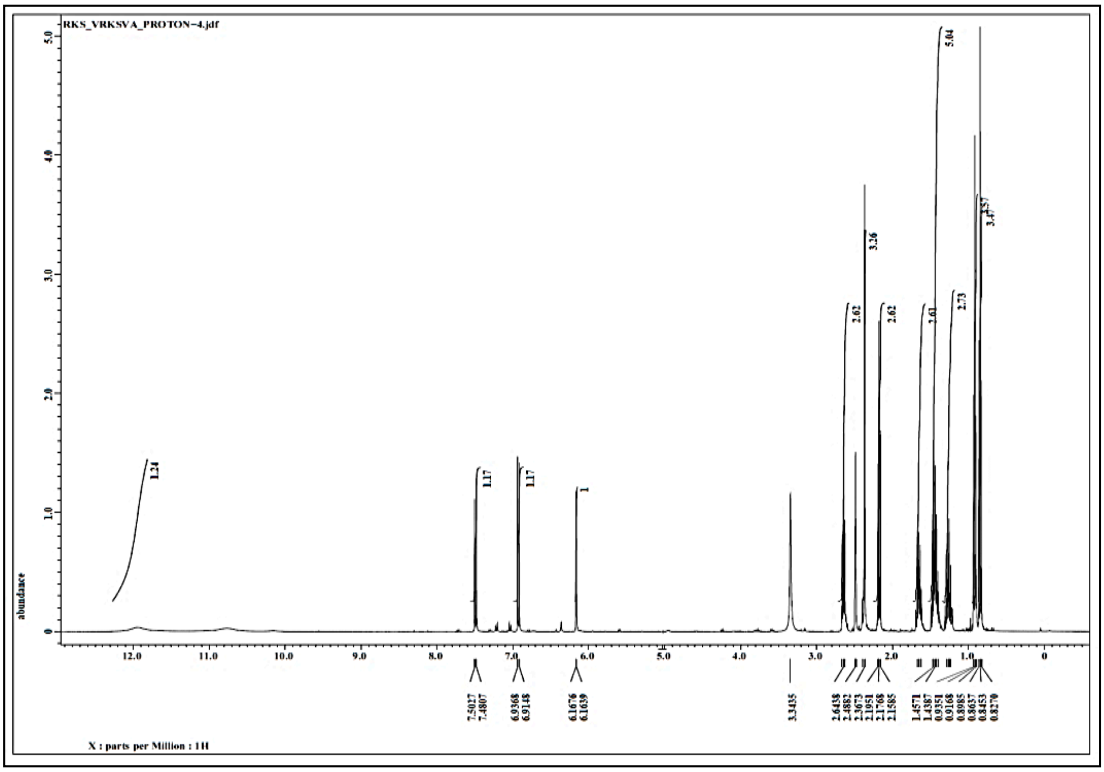

Figure S7. ^{1}H-NMR of 7-Hydroxy-8-pentanoyloxy-4-methylcoumarin (**3d**).

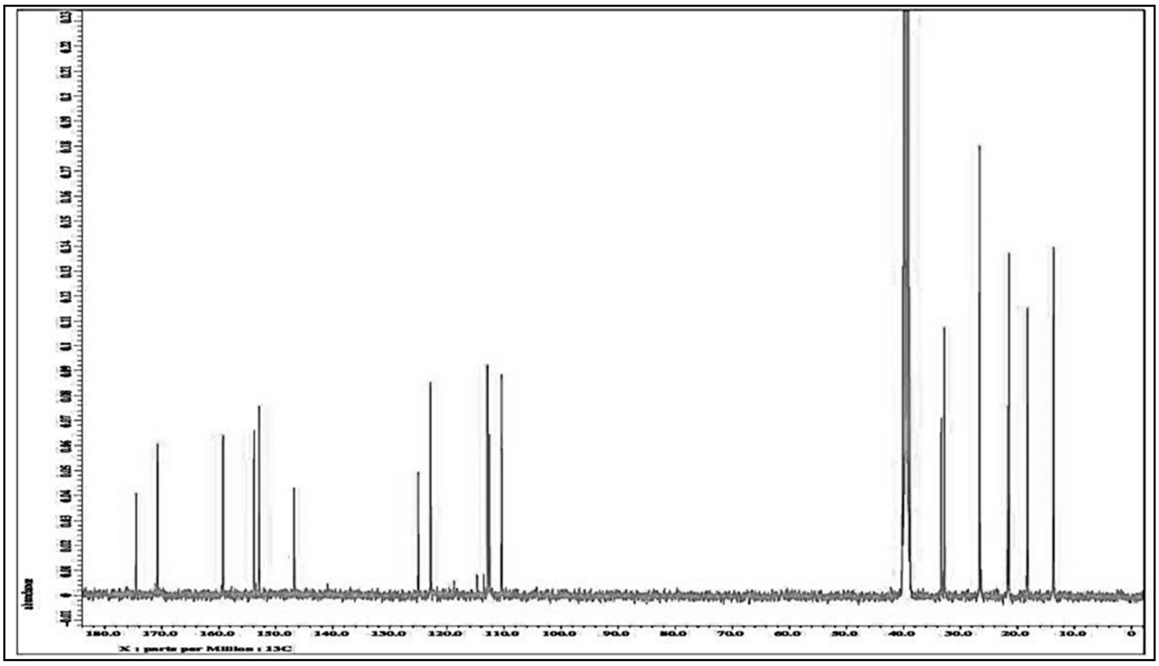

Figure S8. ^{13}C-NMR of 7-Hydroxy-8-pentanoyloxy-4-methylcoumarin (**3d**).

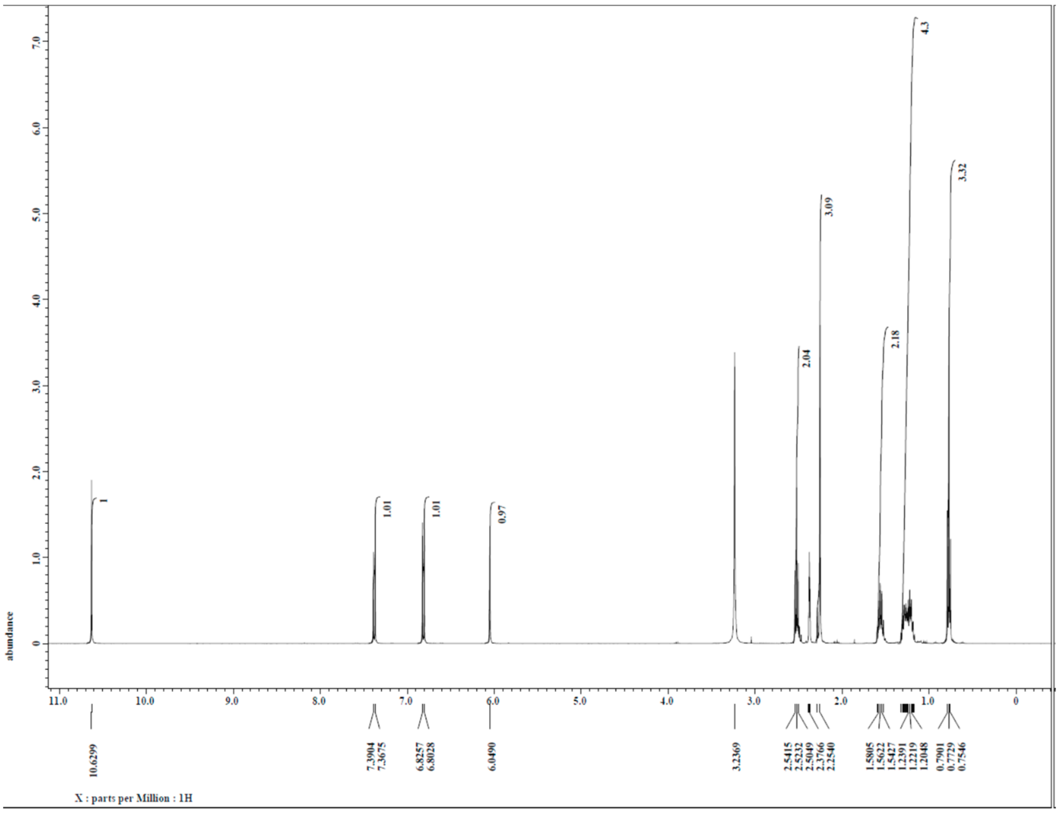

Figure S9. ^{1}H-NMR of 8-Hexanoyloxy-7-hydroxy-4-methylcoumarin (**3e**).

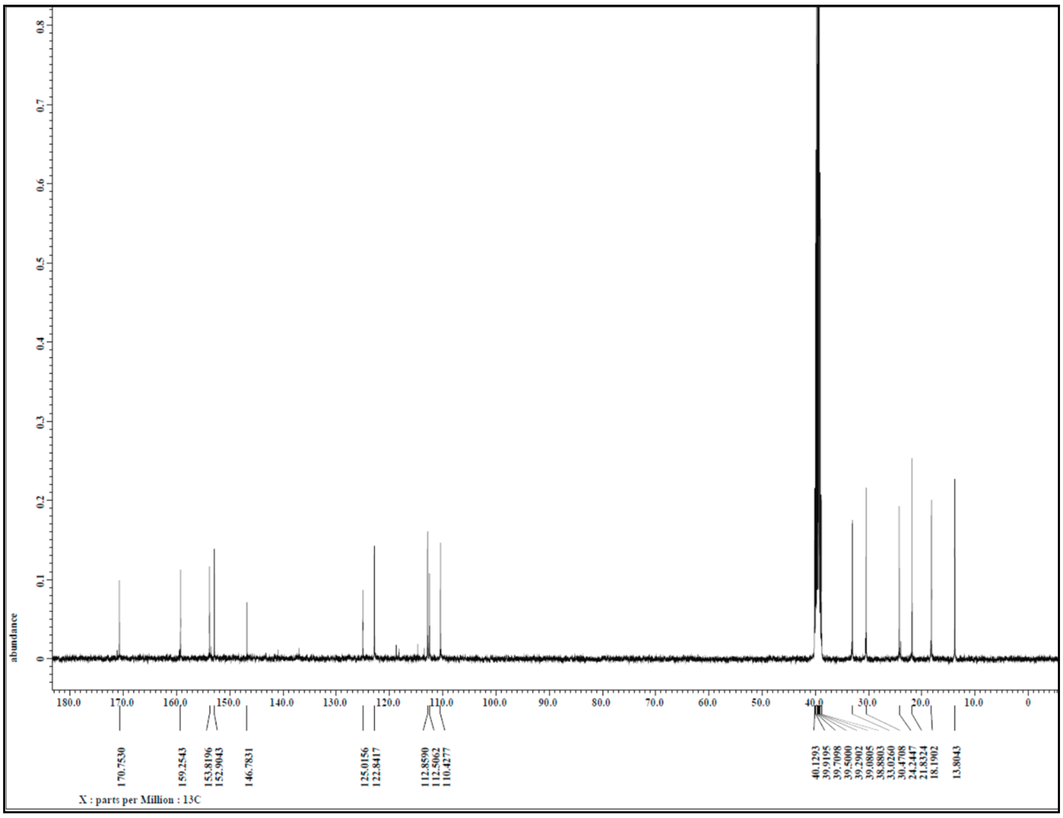

Figure S10. ^{13}C-NMR of 8-Hexanoyloxy-7-hydroxy-4-methylcoumarin (**3e**).

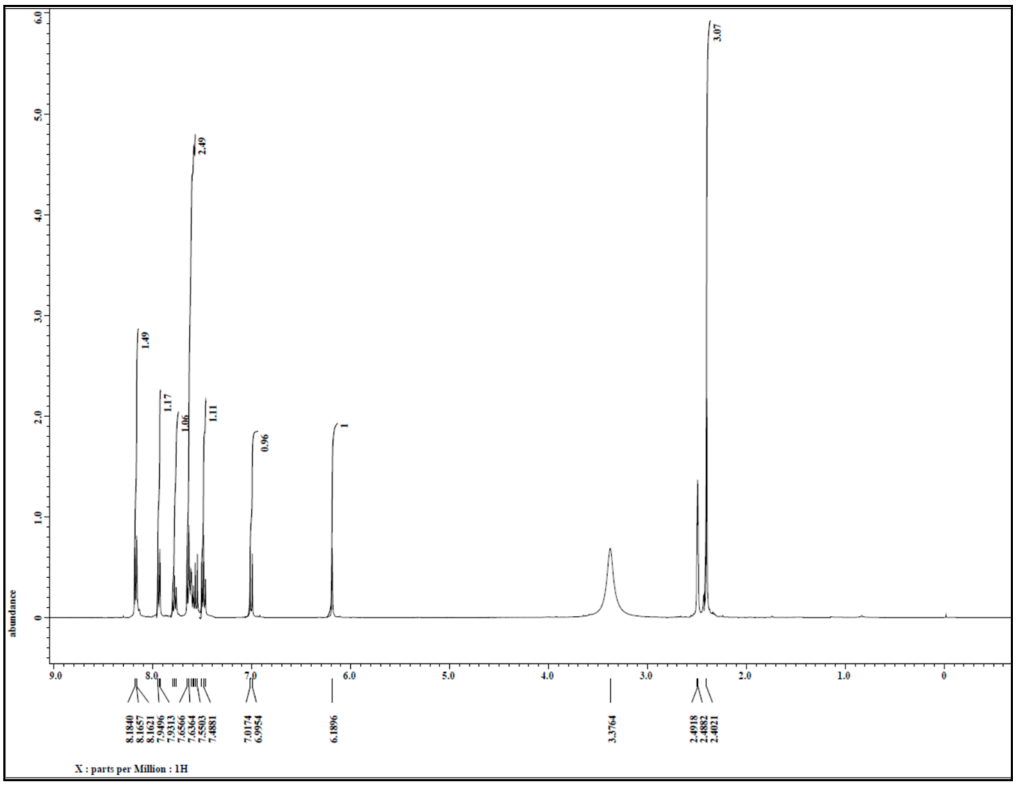

Figure S11. ^{1}H-NMR of 8-Benzoyloxy-7-hydroxy-4-methylcoumarin (**3f**).

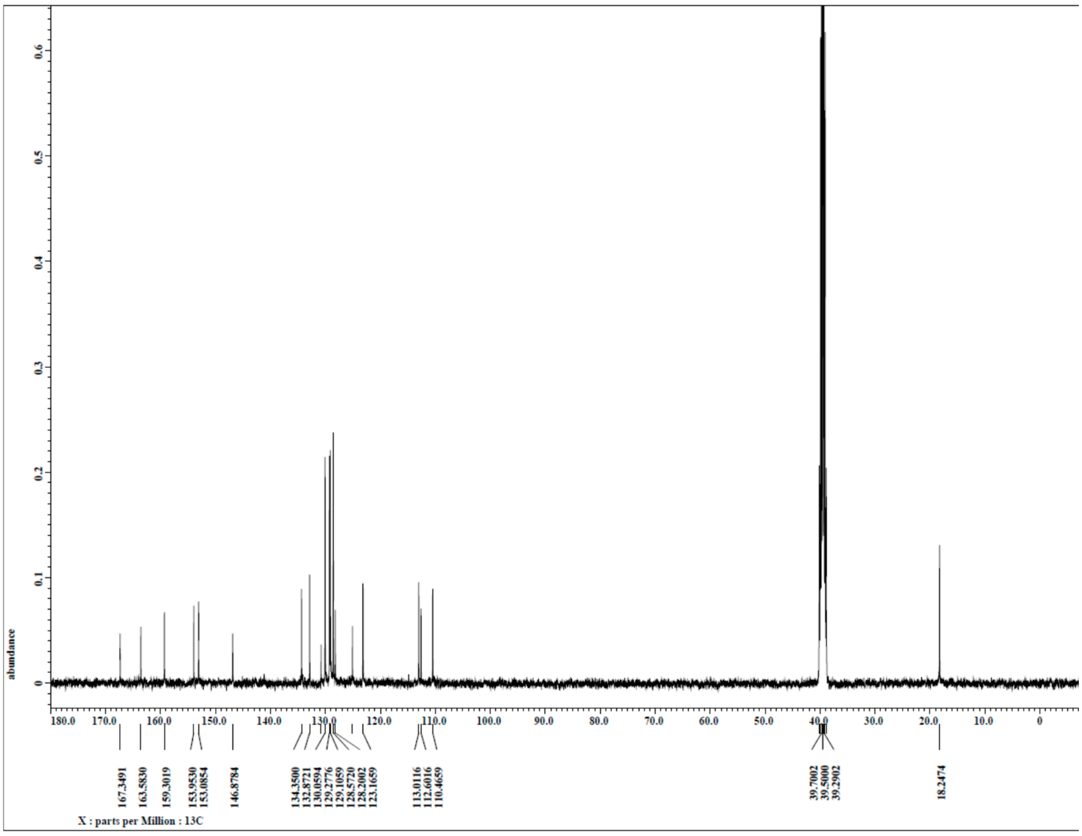

Figure S12. ^{13}C-NMR of 8-Benzoyloxy-7-hydroxy-4-methylcoumarin (**3f**).

2. Single Crystal X-ray Structure and Data of 8-Acetoxy-7-hydroxy-4-methylcoumarin (3a)

Single crystal suitable for X-ray diffraction was grown by dissolving compound **3a** in $CHCl_3$-MeOH mixture and allowing it to evaporate slowly at room temperature. X-ray diffraction data was collected on an Oxford XCalibur CCD diffractometer using graphite monochromated Cu Kα radiation (λ = 0.7107 Å) at temperature 298 K. The structure was solved by direct methods using SIR-92 and refined by full-matrix method (SHELXL-2016/4). All calculations were carried out using the WinGX package of the crystallographic programs. For the molecular graphics, the program Mercury was used. Further information on the crystal structure determination (excluding structure factors) has been deposited in the Cambridge Crystallographic Data Centre as supplementary publications no. 1508122. Molecular structure has been drawn using ORTEP as software as given in Figure S13. The selected bond lengths, bond angles, etc. are given in Table S1.

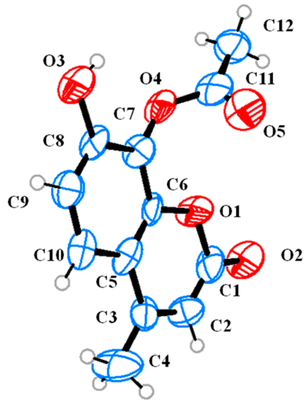

Figure S13. ORTEP diagram showing crystallographic atom numbering and solid-state conformation for 8-acetoxy-7-hydroxy-4-methylcoumarin (**3a**; CCDC-1508122). Crystallographic data have been deposited with the Cambridge Crystallographic Data.

Table S1. Single crystal X-ray diffraction data of compound **3a**.

Compound	3a
Empirical formula	$C_{12}H_{10}O_5$
Formula weight	234.20
Temperature	298(2) K
Wavelength	0.71073 Å
Crystal system	Triclinic
Space group	P 1
Unit cell dimensions	a = 8.1956(18) Å<br>α = 88.259(15) Å<br>b = 11.214(2) Å<br>β = 88.083(16) Å<br>c = 11.8591(19) Å<br>γ = 86.015(18)Å
Volume	1086.2(4) Å
Z	4
Density (calculated)	1.432 Mg/m^3
Absorption coefficient	0.113 mm^{-1}
F(000)	488
Crystal size	0.39 × 0.18 × 0.10 mm^3
Theta range for data collection	2.98°–29.32°
Index ranges	−11 ≤ h ≤ 10<br>−15 ≤ k ≤ 15<br>−15 ≤ l ≤ 15
Reflections collected	10268
Independent reflections	7372 [R(int) = 0.0979]
Completeness to theta = 26.32 °	98.15
Max. and min. transmission	1.0000 and 0.5281
Data/restraints/parameters	7372/3/613
Goodness-of-fit indicator	1.097
Final R indices [I > 2sigma(I)]	R1 = 0.09,<br>wR2 = 0.3040
R indices (all data)	R1 = 0.1238,<br>wR2 = 0.4114
Absolute structure parameter	0.4(10)
Largest diff. peak and hole	0.749 and −0.459 e Å^{-3}
CCDC	1508122
